# Karyotype Variation and Environmental Adaptation in the Invasive Alien Freshwater Flatworm *Girardia* in China

**DOI:** 10.1002/ece3.74095

**Published:** 2026-08-02

**Authors:** Lei Wang, Wei‐Min Ren, Xin‐Xin Sun, De‐Zeng Liu, Zi‐Mei Dong, Guang‐Wen Chen

**Affiliations:** ^1^ College of Life Science Henan Normal University Xinxiang China

**Keywords:** China, ecology, freshwater planarians, global warming, invasive species

## Abstract

Freshwater planarians of the genus *Girardia* demonstrate a remarkable ability to colonize new regions through anthropogenic transport and subsequent natural dispersal. In this study, we conducted a comprehensive field investigation and integrative analysis of *Girardia* species in invaded regions of China. Our results reveal that *G. tigrina* and *G. sinensis* are the principal invasive species in China. Long‐term monitoring indicates a predominance of asexual reproduction, with no evidence of sexual reproduction. Karyological analysis showed a basic haploid complement of 8 metacentric chromosomes in each invasive *Girardia* population. Notably, we identified triploid, mixoploid, and aneuploid individuals—representing the first documentation of such karyotypic diversity in *Girardia* species from China. This variation is consistent with the observed reproductive strategies and may reflect chromosomal instability following introduction. Furthermore, invasive *Girardia* species demonstrate high ecological plasticity, enabling successful colonization and persistence in unfavorable environments, for example, affected by pollution, high temperature, human intervention. Meanwhile they may impact native communities through resource competition and structural shifts. Therefore, the *Girardia* may represent a notable example of how climate change drives the geographic expansion of invasive species. These findings provide crucial baseline data for reconstructing invasion pathways and demographic history, and offer valuable insights for future monitoring, management, and conservation efforts in freshwater ecosystems, as global temperatures rise.

## Graphical abstract

Our findings provide a comprehensive baseline of the distribution, reproductive strategies, and environmental adaptations of invasive Girardia species, and underscore the urgent need for continued monitoring and effective management to safeguard native biodiversity. Considering their broad environmental tolerance and rapid reproductive capacity, together with the accelerating impacts of global warming, proactive management will be essential to limit further spread and mitigate potential ecological disruptions in freshwater ecosystems under a warming climate.

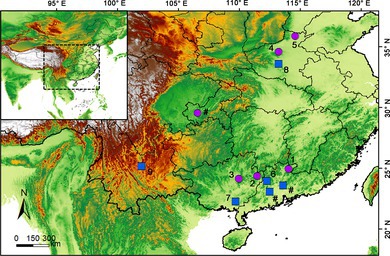

## 1 Introduction

Up to now, approximately 38 species of freshwater planarians have been reported from China, including 20 species of *Dugesia* Girard, 1850, 12 species of *Polycelis* s.l. Ehrenberg, 1831, 3 species each of *Dendrocoelopsis* Kenk, 1930 and *Phagocata* Leidy, 1847, and 2 species of *Gir*ardia Ball, 1974 (Liu 1997; Sun et al. 2024; Wang et al. 2025; Wu et al. 2025, 2026; and references therein). Moreover, recent taxonomic studies have revealed a rich biodiversity of freshwater planarians in China (Wang et al. 2025; Wu et al. 2025).

The genus *Girardia* comprises about 59 valid species, with a natural distribution ranging from southern Argentina to southern Canada (Benítez‐Álvarez, Sluys, et al. 2023). In addition to their native range, *Girardia* species have been introduced to many regions of the world (Stocchino et al. 2019; Benítez‐Álvarez, Leria, et al. 2023 and references therein). Recent research confirms that the *Girardia* populations spreading globally mainly belong to three species: *G. dorotocephala* (Woodworth, 1897), *G. tigrina* (Girard, 1850), and *G. sinensis* Chen & Wang, 2015 (Benítez‐Álvarez, Leria, et al. 2023; Benítez‐Álvarez, Sluys, et al. 2023). The invasive species may raise ecological concerns, particularly in freshwater ecosystems, where they may compete with native species, alter food webs, or serve as intermediate hosts of parasites (Kiruba‐Sankar et al. 2018).

Global warming is increasingly recognized as a key driver of biological invasions in animals (Huang et al. 2011). Rising temperatures can accelerate metabolism, reproduction, and dispersal abilities of invasive alien species, while simultaneously reducing climatic barriers that once limited their survival (Ju et al. 2017). As a result, many invasive species are expanding their ranges into previously unsuitable regions, threatening native biodiversity, altering ecosystem functioning, and intensifying competition with native species (Venette and Hutchison 2021). In particular, the genus *Girardia* demonstrates a remarkable ability to colonize new regions through anthropogenic transport and subsequent natural dispersal (Stocchino et al. 2019; Benítez‐Álvarez, Leria, et al. 2023). Rising water temperatures may further enhance their reproductive success, shorten generation times, and broaden their distribution into temperate zones formerly constrained by thermal limits (Armarego‐Marriott 2025; Dutertre et al. 2010). Thus, species of *Girardia* may represent a notable example of how climate change drives the geographic expansion of invasive species.

However, relatively few studies have been conducted on *Girardia* in China (Chen et al. 2015; Li et al. 2021). Therefore, understanding the identity, distribution, and biological characteristics of invasive *Girardia* is essential for assessing their ecological impact and developing appropriate management strategies. Nevertheless, studies on the life cycle and chromosome karyotype of freshwater *Girardia* species are currently limited and require further investigation, including in China (Stocchino et al. 2019). Information on the life cycle, karyotype, and other biological traits of freshwater *Girardia* species found in China is critical for evaluating their invasive potential and understanding their evolutionary and ecological relationships with native planarians in China.

In this study, we collected *Girardia* specimens from multiple localities across China. Species identification was carried out based on external morphology, molecular analysis, and karyological data. In addition, we investigated their reproductive modes under laboratory conditions to assess their capacity for establishment and potential spread. To date, only *G. sinensis* and *G. tigrina* have been documented in China; these two species exhibit high adaptability (especially for high temperatures) and reproductive plasticity, enabling them to establish populations under a wide range of environmental conditions. Our findings contribute to a better understanding of *Girardia* diversity in China and offer insights into the mechanisms underlying their invasive success in the context of global warming.

## 2 Materials and Methods

### 2.1 Specimen Collection and Culturing

**FIGURE 1:**
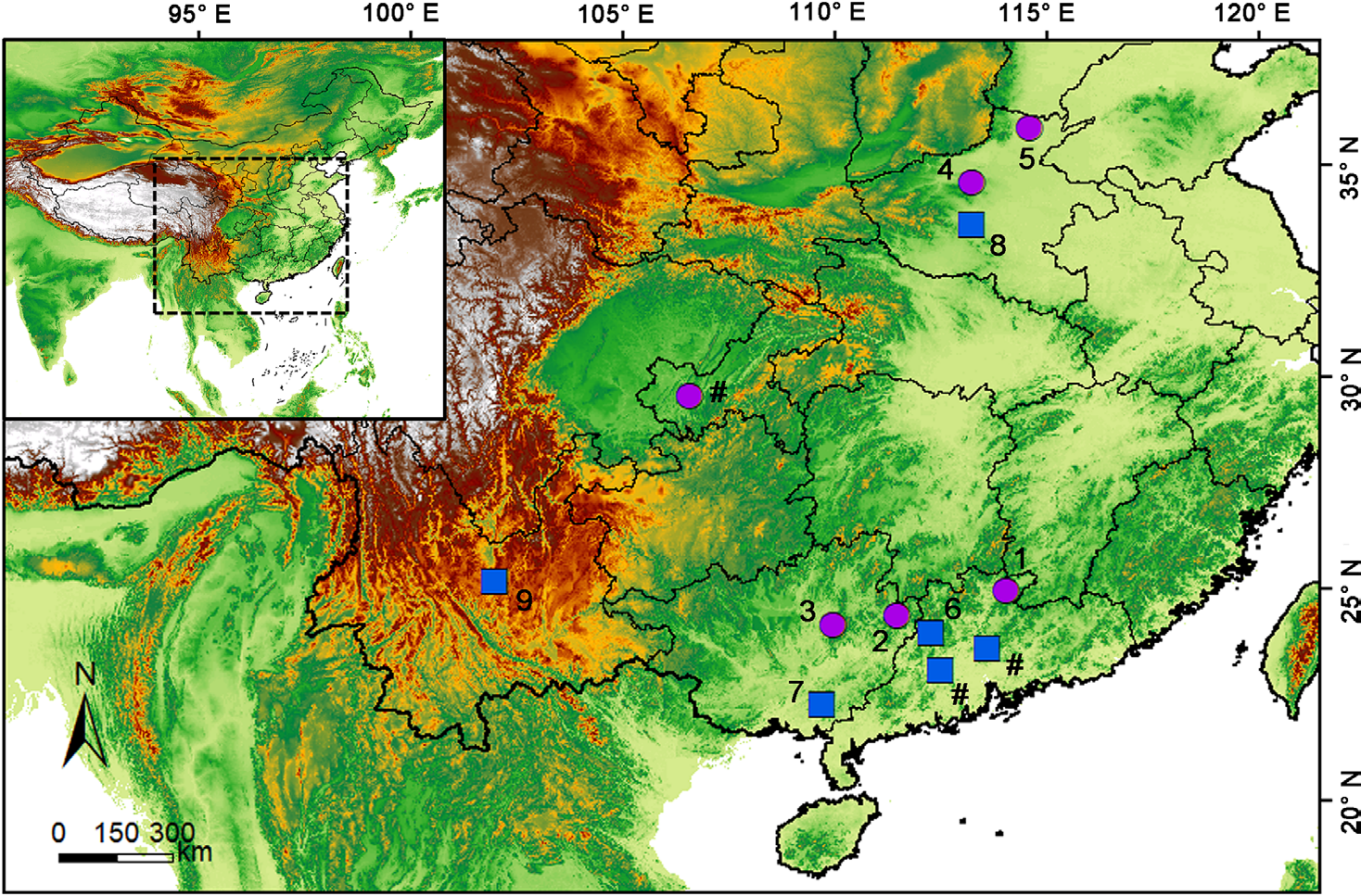
Collection sites of *Girardia* in China. Blue rectangles represent *G. sinensis* , purple circles represent *G. tigrina* , “**#**” indicates previously reported collection sites, and numbers 1–9 indicate the nine sampling sites in the present study.

**FIGURE 2:**
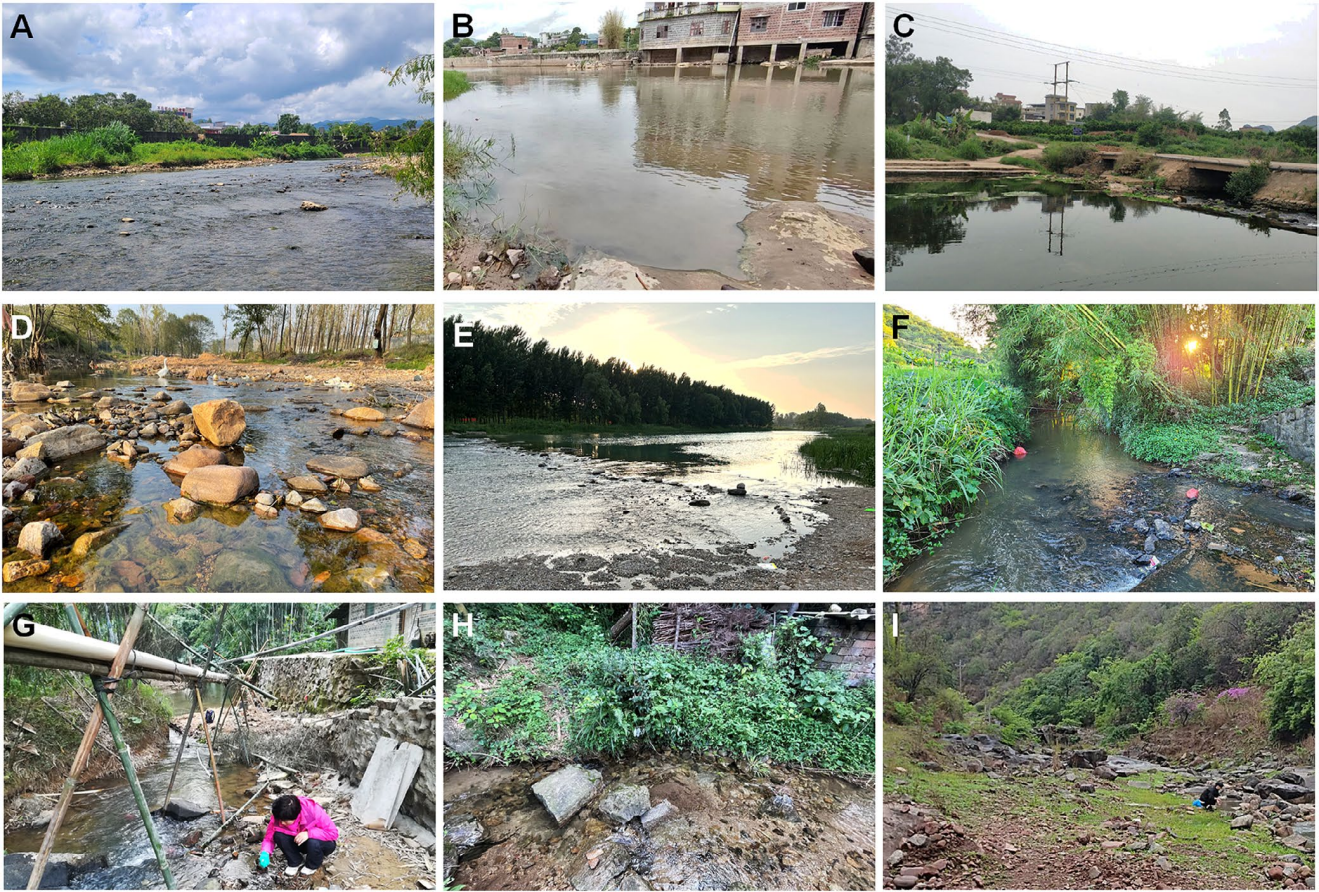
Habitat characteristics of the collection sites for nine *Girardia* populations. (A) *Girardia tigrina* HNLB (No. 1), (B) *Girardia tigrina* MFJB (No. 2), (C) *Girardia tigrina* JXRL (No. 3), (D) *Girardia tigrina* XSRK (No. 4), (E) *Girardia tigrina* QHGY (No. 5), (F) *Girardia sinensis* SZZ (No. 6), (G) *Girardia sinensis* HT (No. 7), (H) *Girardia sinensis* SSD (No. 8), (I) *Girardia sinensis* HMJ (No. 9). The numbers in brackets correspond to the location and habitat numbers shown in Figure 1 and Table 1.

Specimens were collected from under stones in streams or brooks using a paintbrush (The information of collection site and habit see Table 1; Figures 1 and 2). After collection, the worms were transferred to plastic bottles filled with stream water; during transportation to the laboratory, the bottles were placed in a cooler filled with an ice bag.

**TABLE 1:** Collection site information for nine *Girardia* populations.

Number	Species	Population code	Coexistence with *Dugesia*	Locality	Water temperature (°C)	Air temperature	Altitude	Longitude and latitude N/E	Sample time	Sample collectors
1	*Girardia tigrina*	HNLB	Yes	Shaoguan, Guangdong, China	32	34	91 m	N 24°56′58″; E 114°01′22″	2022.08.18	Fan Wu, Yu‐Hao Zhao, and Xiao Liu
2	*Girardia tigrina*	MFJB	Yes	Hezhou, Guangxi, China	28	32	120 m	N 24°20′46″; E 111°28′41″	2022.08.12	Fan Wu, Yu‐Hao Zhao, and Xiao Liu
3	*Girardia tigrina*	JXRL	No	Laibin, Guangxi, China	21	24	15 m	N 24°08′59″; E 109°58′05″	2021.04.09	Zi‐Mei Dong, Yi‐Xuan Wang, and Fan Wu
4	*Girardia tigrina*	XSRK	Yes	Pingdingshan, Henan, China	10	14	560 m	N 34°35′23″; E 113°13′28″	2021.10.24	Lei Wang, Fan Wu, and Fu‐Hao Ma
5	*Girardia tigrina*	QHGY	Yes	Hebi, Henan, China	12	15	10 m	N 35°52′22″; E 114°35′7″	2023.06.10	Lei Wang, Fan Wu, and Dan‐Dan Sun
6	*Girardia sinensis*	SZZ	No	Zhaoqing, Guangdong, China	30	32	98 m	N 23°57′10″; E 112°15′57″	2023.08.14	Fan Wu, Yu‐Hao Zhao, and Xiao Liu
7	*Girardia sinensis*	HT	No	Yulin, Guangxi, China	11	15	99 m	N 22°15′15″; E 109°41′42″	2019.01.03	Zi‐Mei Dong, Lei Wang, and Jin‐Zi Chen
8	*Girardia sinensis*	SSD	No	Pingdingshan, Henan, China	12	16	340 m	N 33°34′23″; E 113°13′28″	2021.04.03	Lei Wang, Fan Wu, and Yu‐Hao Zhao
9	*Girardia sinensis*	HMJ	Yes	Kunming, Yunnan, China	24	26	1569 m	N 25°09′30″; E 101°58′43″	2023.06.15	Fan Wu, Jing‐Long Liu, and Fu‐Hao Ma

In an automatic incubator (BOXUN BSP‐800) the planarians were cultured in autoclaved tap water at 20°C and fed with fresh beef liver once per week. The worms were starved for at least seven days before being used for karyotype and DNA extraction. Images of their external morphology were obtained by using a digital camera attached to a stereo‐dissecting microscope (Leica M165C).

### 2.2 DNA Extraction, Amplification and Sequencing

Procedures for DNA extraction, amplification and sequencing followed Wang et al. (2021). Total genomic DNA was extracted by using the QIAamp DNA Mini Tissue Kit (Qiagen, Germany), according to the manufacturer's protocols. The quality and quantity of DNA were determined by NanoDrop oneC (Thermo Scientific). For each population, fragments of Cytochrome Oxidase I (*COI*) and internal Elongation factor 1 alpha (*EF1α*) genes were amplified. Primers used for amplification and the PCR protocol were by Benítez‐Álvarez, Sluys, et al. (2023). Premix Ex Taq Hot Start Version (TaKaRa, Otsu Japan) was used for the polymerase chain reaction (PCR). Amplifications were conducted in a final volume of 30 μL under the following conditions: 5 min at 94°C, 35 cycles of 40s at 94°C, an annealing step for 30s, and 1 min at 72°C, and 5 min at 72°C as a final extension. Purification of PCR products and sequencing were done by GENEWIZ (Tianjin, China). Sequencing reactions were performed with the same primers used to amplify the fragments. All specimens were sequenced for both forward and reverse DNA strands.

### 2.3 Phylogenetic Analysis and Genetic Distances

The datasets comprised newly obtained *EF1α* and *COI* sequences from *G. tigrina* and *G. sinensis* specimens, together with available sequences of *Girardia* representatives from Asia, Australia, Europe, and the Americas, were used to perform phylogenetic analyses (Table 2). The *Girardia* trees were rooted on the node separating the clade constituted by *Girardia schubarti* (Marcus 1946) and a group of North American species from the rest, following the results of Benítez‐Álvarez, Sluys, et al. (2023).

**TABLE 2:** GenBank accession numbers of sequences used in molecular analyses.

Code in the tree	Species	GenBank	
		*COI*	*EF1α*
*Girardia anderlani* Brazil 1	*Girardia anderlani*	OM232748.1	—
*Girardia anderlani* Brazil 2	*Girardia anderlani*	DQ666038.1	—
*Girardia arenicola* Brazil	*Girardia arenicola*	OM264750	OM418632
*Girardia biapertura* Brazil	*Girardia biapertura*	OM307082	—
*Girardia multidiverticulata* Brazil 1	*Girardia multidiverticulata*	OM307125	OM418643
*Girardia multidiverticulata* Brazil 2	*Girardia multidiverticulata*	OM307109	OM418642
*Girardia multidiverticulata* Brazil 3	*Girardia multidiverticulata*	OM307123	OM418641
*Girardia clandestina* Brazil 1	*Girardia clandestina*	OM307110	OM418692
*Girardia clandestina* Brazil 2	*Girardia clandestina*	OM307085	OM418690
*Girardia clandestina* Brazil 3	*Girardia clandestina*	OM307086	OM418689
*Girardia clandestina* Brazil 4	*Girardia clandestina*	OM307134	OM418686
*Girardia clandestina* Brazil 5	*Girardia clandestina*	OM307081	OM418688
*Girardia tomasi* Argentina 1	*Girardia tomasi*	MW271864	—
*Girardia tomasi* Argentina 2	*Girardia tomasi*	MW271868	—
*Girardia tomasi* Argentina 3	*Girardia tomasi*	MW271863	—
*Girardia sanchezi* Chile 1	*Girardia sanchezi*	OM307168.1	OM418645
*Girardia sanchezi* Chile 2	*Girardia sanchezi*	OM307167.1	OM418644
*Girardia somuncura* Argentina 1	*Girardia somuncura*	MW271865	—
*Girardia somuncura* Argentina 2	*Girardia somuncura*	MW271866	—
*Girardia somuncura* Argentina 3	*Girardia somuncura*	MW271867	—
*Girardia somuncura* Argentina 4	*Girardia somuncura*	MW271869	—
*Girardia schubarti* Brazil 1	*Girardia schubarti*	OM307131	OM418646
*Girardia schubarti* Brazil 2	*Girardia schubarti*	OM307080	OM418649
*Girardia schubarti* Brazil 3	*Girardia schubarti*	OM307079	OM418648
*Girardia schubarti* Brazil 4	*Girardia schubarti*	—	OM418651
*Girardia schubarti* Brazil 5	*Girardia schubarti*	DQ666041.1	KJ599691.1
*Girardia schubarti* Brazil 6	*Girardia schubarti*	OM307131	OM418646
*Girardia dorotocephala* USA 1	*Girardia dorotocephala*	OM307147	OM349496
*Girardia dorotocephala* USA 2	*Girardia dorotocephala*	OM307112	OM349498
*Girardia dorotocephala* USA 3	*Girardia dorotocephala*	OM307113	OM349499
*Girardia dorotocephala* USA 4	*Girardia dorotocephala*	OM307124	OM349489
*Girardia dorotocephala* USA 5	*Girardia dorotocephala*	OM307136	OM349491
*Girardia dorotocephala* USA 6	*Girardia dorotocephala*	OM307138	OM349502
*Girardia dorotocephala* USA 7	*Girardia dorotocephala*	OM307140	OM349492
*Girardia dorotocephala* USA 8	*Girardia dorotocephala*	OM307145	OM349495
*Girardia dorotocephala* USA 9	*Girardia dorotocephala*	OM307147	OM349496
*Girardia dorotocephala* Mexico 1	*Girardia dorotocephala*	OM307177	OM349487
*Girardia dorotocephala* Mexico 2	*Girardia dorotocephala*	OM307151	OM349497
*Girardia dorotocephala* Mexico 3	*Girardia dorotocephala*	OM307150	—
*Girardia dorotocephala* Mexico 4	*Girardia dorotocephala*	OM307177	OM349487
*Girardia dorotocephala* Mexico 5	*Girardia dorotocephala*	OM307179	—
*Girardia dorotocephala* France	*Girardia dorotocephala*	OM307073	OM349486
*Girardia dorotocephala* spain	*Girardia dorotocephala*	OM307103	OM349494
*Girardia dorotocephala* Portugal	*Girardia dorotocephala*	OM307104	OM349490
*Girardia dorotocephala* Brazil	*Girardia dorotocephala*	OM307111	OM349488
*Girardia dorotocephala* Hawaii 1	*Girardia dorotocephala*	OM307129	OM349501
*Girardia dorotocephala* Hawaii 2	*Girardia dorotocephala*	OM307130	OM349500
*Girardia dorotocephala* Japan	*Girardia dorotocephala*	OM307166	OM349493
*Girardia tigrina* Spain 1	*Girardia tigrina*	OM307091	OM418681
*Girardia tigrina* Spain 2	*Girardia tigrina*	OM307077	OM418675
*Girardia tigrina* Spain 3	*Girardia tigrina*	OM307078	OM418673
*Girardia tigrina* Spain 4	*Girardia tigrina*	OM307092	—
*Girardia tigrina* Spain 5	*Girardia tigrina*	OM307127	OM418680
*Girardia tigrina* Spain 6	*Girardia tigrina*	OM307141	OM418670
*Girardia tigrina* Spain 7	*Girardia tigrina*	OM307142	OM418684
*Girardia tigrina* Spain 8	*Girardia tigrina*	OM307149	—
*Girardia tigrina* Spain 9	*Girardia tigrina*	OM307156	OM418685
*Girardia tigrina* Spain 10	*Girardia tigrina*	DQ666042	—
*Girardia tigrina* Spain 11	*Girardia tigrina*	AF178316	—
*Girardia tigrina* France 1	*Girardia tigrina*	OM307076	OM418671
*Girardia tigrina* France 2	*Girardia tigrina*	OM307087	OM418677
*Girardia tigrina* France 3	*Girardia tigrina*	—	OM418679
*Girardia tigrina* France 4	*Girardia tigrina*	OM307088	OM418682
*Girardia tigrina* France 5	*Girardia tigrina*	OM307105	OM418674
*Girardia tigrina* France 6	*Girardia tigrina*	DQ666042.1	—
*Girardia tigrina* Italy 1	*Girardia tigrina*	OM307101	OM418683
*Girardia tigrina* Germany 1	*Girardia tigrina*	OM307144	OM418678
*Girardia tigrina* Germany 2	*Girardia tigrina*	OM307154	—
*Girardia tigrina* Canada 1	*Girardia tigrina*	OM307160	OM418676
*Girardia tigrina* USA	*Girardia tigrina*	OM307163	OM418672
*Girardia tigrina* China 1	*Girardia tigrina*	MT992025	—
*Girardia tigrina* XSRK^a^	*Girardia tigrina*	PZ482336	PZ441343
*Girardia tigrina* QHGY^a^	*Girardia tigrina*	PZ482337	PZ441341
*Girardia tigrina* JXRL^a^	*Girardia tigrina*	PZ482338	PZ441339
*Girardia tigrina* HNLB^a^	*Girardia tigrina*	PZ482339	PZ441336
*Girardia tigrina* MFJB^a^	*Girardia tigrina*	PZ482340	PZ441340
*Girardia sinensis* Morocco 1	*Girardia sinensis*	OM480692	OM487088
*Girardia sinensis* Morocco 2	*Girardia sinensis*	OM480693	OM487089
*Girardia sinensis* Morocco 3	*Girardia sinensis*	OM480694	OM487090
*Girardia sinensis* China 1	*Girardia sinensis*	KPO91895	—
*Girardia sinensis* China 2	*Girardia sinensis*	OM307169	OM418662
*Girardia sinensis* Spain 1	*Girardia sinensis*	OM307098	OM418657
*Girardia sinensis* Spain 2	*Girardia sinensis*	OM307089	OM418669
*Girardia sinensis* Spain 3	*Girardia sinensis*	OM307159	—
*Girardia sinensis* Spain 4	*Girardia sinensis*	OM418668	OM307158
*Girardia sinensis* Spain 5	*Girardia sinensis*	OM307157	—
*Girardia sinensis* Spain 6	*Girardia sinensis*	OM307128	OM418658
*Girardia sinensis* Spain 7	*Girardia sinensis*	OM307106	OM418654
*Girardia sinensis* Spain8	*Girardia sinensis*	OM307102	OM418653
*Girardia sinensis* Spain 9	*Girardia sinensis*	OM307097	—
*Girardia sinensis* Spain 10	*Girardia sinensis*	OM307096	OM418663
*Girardia sinensis* Spain 11	*Girardia sinensis*	OM307090	OM418664
*Girardia sinensis* Spain 12	*Girardia sinensis*	OM307093	—
*Girardia sinensis* Spain 13	*Girardia sinensis*	OM307094	—
*Girardia sinensis* Spain 14	*Girardia sinensis*	OM307095	—
*Girardia sinensis* ltaly 1	*Girardia sinensis*	OM307099	OM418655
*Girardia sinensis* ltaly 2	*Girardia sinensis*	OM307100	—
*Girardia sinensis* Netherlands 1	*Girardia sinensis*	OM307155	OM418665
*Girardia sinensis* Australia 1	*Girardia sinensis*	OM307161	OM418659
*Girardia sinensis* Australia 2	*Girardia sinensis*	OM307162	OM418660
*Girardia sinensis* Australia 3	*Girardia sinensis*	OM307146	OM418667
*Girardia sinensis* France 1	*Girardia sinensis*	OM307152	—
*Girardia sinensis* France 2	*Girardia sinensis*	OM307121	OM418656
*Girardia sinensis* France 3	*Girardia sinensis*	OM307122	—
*Girardia sinensis* Cuba 1	*Girardia sinensis*	OM307174	OM418661
*Girardia sinensis* Germany 1	*Girardia sinensis*	OM307153	OM418666
*Girardia sinensis* Portugal 1	*Girardia sinensis*	OQ185384	OM418681
*Girardia sinensis* HT^a^	*Girardia sinensis*	PZ482332	PZ441338
*Girardia sinensis* HMJ^a^	*Girardia sinensis*	PZ482333	PZ441344
*Girardia sinensis* SSD^a^	*Girardia sinensis*	PZ482334	PZ441342
*Girardia sinensis* SZZ^a^	*Girardia sinensis*	PZ482335	PZ441337
*Girardia* sp. 1_1	*Girardia* sp. 1	OM307171	OM418640
*Girardia* sp. 1_2	*Girardia* sp. 1	OM307172	—
*Girardia* sp. 1_3	*Girardia* sp. 1	MN652340.1	—
*Girardia* sp. 1_4	*Girardia* sp. 1	MN652378.1	—
*Girardia* sp. 1_5	*Girardia* sp. 1	—	OM418694
*Girardia* sp. 1_6	*Girardia* sp. 1	—	OM418639
*Girardia* sp. 2	*Girardia* sp. 2	OM307173	OM418633
*Girardia* sp. 3	*Girardia* sp. 3	OM307132	—
*Girardia* sp. 4	*Girardia* sp. 4	OM307175	OM418634
*Girardia* sp. 5_1	*Girardia* sp. 5	OM307114	OM418636
*Girardia* sp. 5_2	*Girardia* sp. 5	OM307118	OM418638
*Girardia* sp. 5_3	*Girardia* sp. 5	OM307119	OM418637
*Girardia* sp. 6	*Girardia* sp. 6	OM307133	OM418635
*Girardia* sp. 7	*Girardia* sp. 7	OM307176	OM418693

^a^ The new specimens of this study.

Protein‐coding sequences (*EF1α* and *COI*) were translated into amino acid sequences to check for the presence of stop codons. Subsequently, sequences were aligned online with Translator X (Abascal et al. 2010; http://translatorx.co.uk) using the FFT‐NS‐2 method, checked with BioEdit 7.2.6.1 (Hall 1999), and thereafter, back‐translated to nucleotide sequences. Since automated removal of gap columns and variable regions has been reported to negatively affect the accuracy of the inferred phylogeny (Dessimoz and Gil 2010; Tan et al. 2015), the Gblocks option was disabled (Talavera and Castresana 2007). A total of three datasets were used in this study, viz., dataset I: *COI*, dataset II: *EF1α*, dataset III: concatenated sequences *COI* + *EF1α*. The substitution saturation test for *COI* (using DAMBE 6, according to Xia 2018) showed evidence of saturation.

Bayesian information criterion (BIC) was implemented in PartitionFinder 2 (Lanfear et al. 2017) to estimate the best‐fit partition schemes and models of each gene. The evolutionary models were set to GTR + G + I for all datasets and partitions with unlinked parameters estimation. Bayesian inference analysis (BI) was run with MrBayes v3.2 (Ronquist et al. 2012) using two replicate runs with four chains for 3 million generations, sampling trees every 1000 generations. The convergence of runs was checked by monitoring that the standard deviation of split frequencies reached a value below 0.01, thus indicating that the runs had reached a stationary state. Following completion of each analysis, the first 25% of the generated trees were discarded as “burn‐in”. Maximum likelihood (ML) analysis with IQ‐TREE (Minh et al. 2020) was used to perform 10,000 replicates of ultrafast bootstrap approximation (Hoang et al. 2018). BI and ML trees were visualized and edited using Figtree v1.4.3.

Genetic distances based on dataset I and dataset II were calculated by MEGA 7.0 (Kumar et al. 2016) with the Kimura 2‐parameter substitution model (Solà et al. 2013). In order to obtain more accurate values for the genetic distances, the very short sequences were removed, since the study of Marques et al. (2018) indicated that at least 600 bp of *COI* needs to be used in the determination of interspecific divergences and for species delineation, at least in the land planarian genus *Obama* Carbayo et al. (2013). Subsequently, the resulting distance matrices were visualized with the “ggplot2” package in R software (available at https://www.R‐project.org).

### 2.4 Karyology

Air‐drying method was used to obtain karyological preparations, according to the protocols described by Wang et al. (2024). In brief, 6–10 individuals were randomly selected (from each population), with each of the worms being treated separately. Regenerating blastemas were obtained by transversely cutting each worm into 3 fragments (head, middle, tail), which were cultured for 3 days. After 3 days, 4 blastemas (head, prepharyngeal, post‐pharyngeal, tail) were cut off transversely and placed on 4 separate slides and thereafter, treated with a 0.02% colchicine solution at 4°C for 3 h. Hereafter, the blastemas were placed in 0.1% KCl hypotonic solution for 3 h. Subsequently, the surplus hypotonic solution was removed from the slides, and then fixative fluid I, II, III were added; the blastemas were evenly hammered with an oversized needle in order to create a cell suspension (spread over the glass slide). After 24 h (dried at room temperature), the slides were stained for 15 min with a 0.5% Giemsa solution, whereafter the slides were washed with deionized water and dried at room temperature. Hereafter, coverslips were attached with DPX mountant (MFD1266, MesGen Biotechnology Co. Ltd., Shanghai, China).

Mitotic metaphase chromosomes were observed and photographed under a compound microscope (ZEISS, Axio Scope. A1) equipped with a CoolCube digital camera (MetaSystems, Altlussheim, Germany). Karyograms were prepared using the IKAROS Karyotyping system (MetaSystems, Altlussheim, Germany). Well‐spread sets of metaphase plates from randomly selected individuals were used to determine ploidy level, centromeric indices, and relative lengths of the chromosomes. Hereafter, relative lengths of the chromosomes and centromeric positions were re‐checked by manual calibration. In the calculations of the averages, the very few heteromorphic chromosomes and karyotypes were not included in order to avoid misleading results and incorrect conclusions. Karyotype parameter measurements were executed as published previously by Chen et al. (2008). Chromosomal nomenclature followed Levan et al. (1964).

## 3 Results

### 3.1 Morphological Description

**FIGURE 3:**
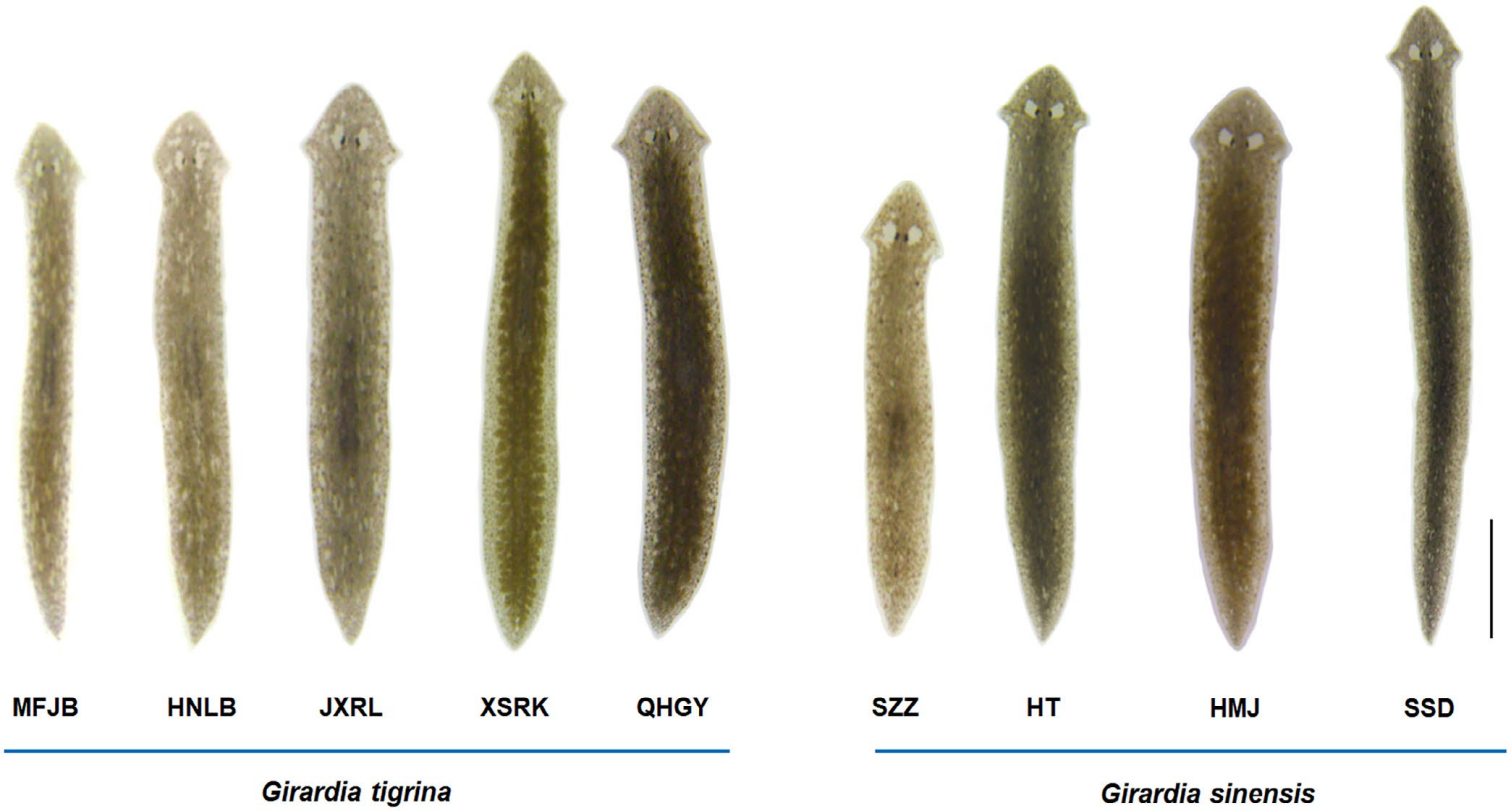
External appearance of nine *Girardia* populations. Scale bar: 2 mm.

All newly obtained populations exhibited the common morphological characteristics of *Girardia*. The triangular head bears two eyes, which are located in the middle of the head at the level of auricles, positioned close together and placed in pigment‐free spots. Unpigmented auricular grooves are marginally situated just posterior to the eyes (Figure 3).

Body size of living asexual individuals is listed in Table 3. Owning to great morphological similarity among *Girardia* species, and because the worms reproduce only asexually by fissiparity (do not exhibit copulatory apparatus), the traditional taxonomic identification is not feasible in this case. Therefore, we use DNA barcoding and molecular phylogenetic analysis to identify the species.

**TABLE 3:** Morphological and karyotypic data of nine *Girardia* populations.

Species	Population code	Body length BL (mm)	Pharynx length PL (mm)	Body width BW (mm)	Pharynx length/Body length PL/BL	Karyotype
*Girardia tigrina*	MFJB	7.51 ± 1.57	1.33 ± 0.34	0.78 ± 0.20	0.18	Mixoploid: 2*n* = 2*x* = 16 (54) & 2*n* = 3*x* = 24 (86)
*Girardia tigrina*	HNLB	7.71 ± 1.40	1.25 ± 0.16	0.92 ± 0.18	0.16	Mixoploid: 2*n* = 2*x* = 16 (39) & 2*n* = 3*x* = 24 (110)
*Girardia tigrina*	XSRK	9.98 ± 1.67	1.82 ± 0.35	1.08 ± 0.26	0.18	Mixoploid: 2*n* = 2*x* = 16 (51) & 2*n* = 3*x* = 24 (91)
*Girardia tigrina*	QHGY	8.76 ± 1.67	1.61 ± 0.25	0.78 ± 0.34	0.18	Triploid: 3*n* = 3*x* = 24 (151)
*Girardia tigrina*	JXRL	9.71 ± 1.40	1.87 ± 0.40	1.11 ± 0.18	0.19	Triploid and aneuploid: 2*n* = 3*x* = 24 (99) & 2*n* = 3*x* = 24‐18th (40) & 2*n* = 3*x* = 24‐28th (24)
*Girardia sinensis*	HT	9.05 ± 1.74	1.45 ± 0.60	1.08 ± 0.40	0.16	Mixoploid: 2*n* = 2*x* = 16 (83) & 2*n* = 3*x* = 24 (73)
*Girardia sinensis*	SZZ	5.58 ± 0.37	1.13 ± 0.21	0.72 ± 0.11	0.20	Mixoploid: 2*n* = 2*x* = 16 (81) & 2*n* = 3*x* = 24 (98)
*Girardia sinensis*	SSD	10.26 ± 1.67	1.73 ± 0.20	0.98 ± 0.23	0.17	Mixoploid: 2*n* = 2*x* = 16 (50) & 2*n* = 3*x* = 24 (62)
*Girardia sinensis*	HMJ	10.67 ± 1.67	1.95 ± 0.23	0.56 ± 0.24	0.18	Mixoploid: 2*n* = 2*x* = 16 (86) & 2*n* = 3*x* = 24 (94)

*Note:* The numbers shown in the brackets indicate the number of metaphase plates.

### 3.2 Molecular Phylogeny and Genetic Distances

Phylogenetic trees were constructed using the *EF1α* sequences (834 bp; dataset I), *COI* sequences (762 bp; dataset II), and concatenated sequence (1596 bp; dataset III), respectively.

**FIGURE 4:**
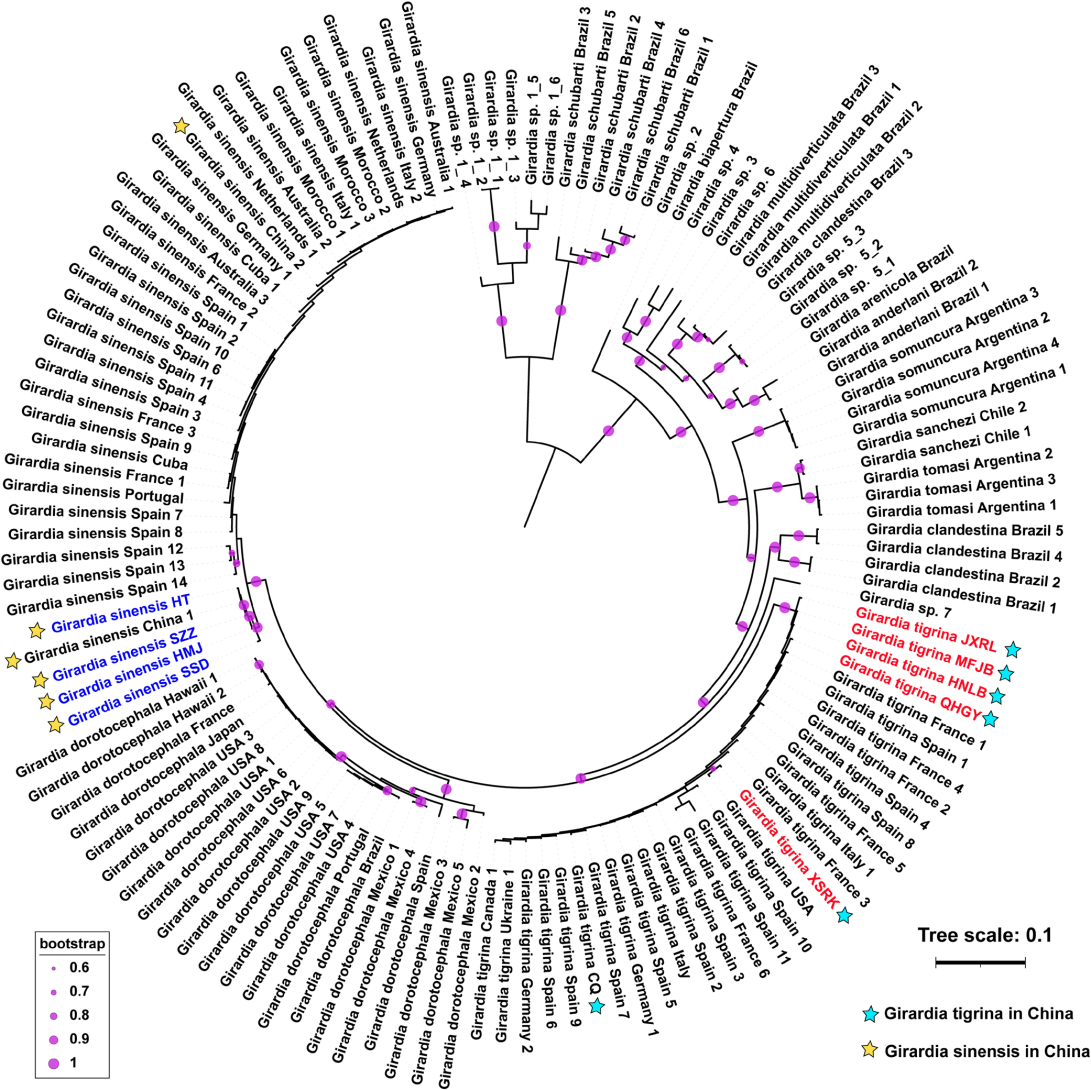
Molecular phylogenetic tree obtained from BI analysis of the dataset III. Pink circle at nodes indicate support values (bs). New obtained *Girardia tigrina* indicated in red, new obtained *Girardia sinensis* indicated in blue. Yellow asterisks indicate the current populations of *G. sinensis* in China, while blue asterisks indicate *G. tigrina* in China. Scale bar: Substitutions per site.

The trees generated by Bayesian Inference (BI) and Maximum Likelihood (ML) analyses showed similar topologies, differing only in a few branches and support values (Figures 4 and S1). According to the recent studies on the phylogenetic relationships within the genus *Girardia* (Benítez‐Álvarez, Leria, et al. 2023; Benítez‐Álvarez, Sluys, et al. 2023; Catalá et al. 2024), the trees were rooted on *G. schubarti* together with *Girardia* sp. 1 (unidentified specimens) from Mexico & USA. Consistent with findings from previous studies, in our phylogenetic analysis each identifed species and the seven delimited unidentifed individuals (obtained from Mexico, Brazil, and Chile, *Girardia* sp. 1–7) recover monophyletic groups, respectively. Notably, all non‐American specimens cluster within the three invasive species, viz. *G. sinensis* , *G. dorotocephala* and *G. tigrina* (together with American individuals), each forming a monophyletic clade. The three species show close relationship. The *G. sinensis* and *G. dorotocephala* form a larger clade, which shares a sister–group relationship with *G. tigrina* . Together, the three species are sister to *Girardia* sp. 7 (an unidentifed species from Mexico). The newly obtained specimens fall into two clades, *G. tigrina* and *G. sinensis* (Figures 4 and S1). Significantly, the specimens HT, SZZ, HMJ and SSD are assigned into *G. sinensis* (pp = 1.00), which comprises individuals from Morocco, China, Spain, Italy, The Netherlands, Australia, France, Cuba, Germany, and Portugal. Meanwhile, specimens JXRL, MFJB, HNLB, QHGY and XSRK from China fall into *G. tigrina* (pp = 1.00), which comprises individuals from Spain, France, Italy, Germany, USA and China.

**FIGURE 5:**
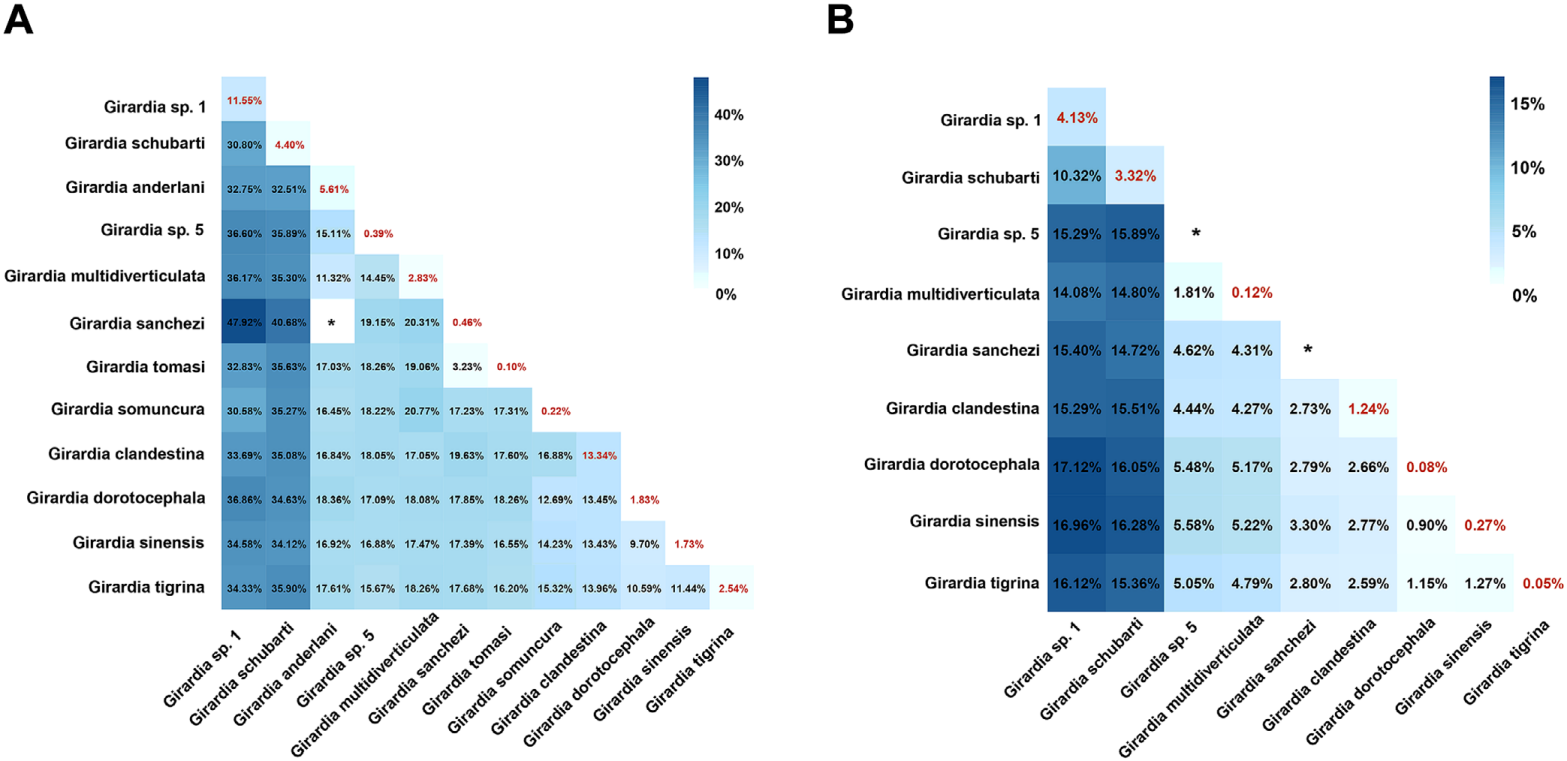
Genetic distance within and between groups among different species of *Girardia* genus. (A) *COI*, (B) *EF1α* (“*” indicates that only one sequence is available or the sequence is too short to calculate).

The mean interspecific genetic distances between the studied *Girardia* species were calculated based on the mitochondrial (*COI*) and nuclear (*EF1α*) genes (Figure 5A,B). For the *COI*, the mean interspecific distances ranged from 9.7% (between *G. sinensis* and *G. dorotocephala* ) to 47.92% (between *G. sanchezi* and *Girardia* sp. 1). For the *EF1α*, interspecific distances ranged from 0.90% (between *G. sinensis* and *G. dorotocephala* ) to 17.12% (between *G. dorotocephala* and *Girardia* sp. 1). In general, interspecific genetic distances exceeded intraspecific distances, supporting the genetic distinctiveness of most species included in this study, although a few exceptions were observed, including *Girardia* sp. 1 (*COI*) and in *G. schubarti* , *G. clandestina* , and *Girardia* sp. 1 (*EF1α*), where intraspecific distances showed relatively high variation.

Among individuals of *G. sinensis* , the average intraspecific genetic distance based on *COI* was 1.73%, and 0.27% based on *EF1α*. The maximum intraspecific genetic distances were 3.04% (*COI*) and 0.94% (*EF1α*), respectively (Figure 5; Tables S1 and S2). With respect to *G. tigrina* , the average intraspecific genetic distances were 2.54% (*COI*) and 0.05% (*EF1α*), with corresponding maximum values of 2.96% and 0.56%, respectively. For *G. dorotocephala* , the average intraspecific genetic distances were 1.83% (*COI*) and 0.08% (*EF1α*), with corresponding maximum values of 5.29% and 0.37%, respectively (Figure 5; Tables S1 and S2).

### 3.3 Collection Sites and Invasive Habitats, Reproduction

#### 3.3.1 *Girardia tigrina* HNLB

The animals were collected from under stones in a river near the village Shixing County, Shaoguan City, Guangdong Province, China (Figure 2A and Table 1). The river is close to a village road. Both banks were thickly covered with grasses, and a few traces of human activity were evident. The current was fast flowing, and a large amount of moss or aquatic plants attached to the stones in the river. *Girardia* and *Dugesia* were found at the same collection site. Under the stereo‐microscope, the worms were sorted based on general morphology. Approximately 24 specimens were identified as *Girardia*, all of which were asexual. *G. tigrina* HNLB, has been maintaied under laboratory conditions for three years. Throughout this period, the individuals reproduced exclusively by fission (with division occurring slightly behind the pharynx), and the final population size exceeded 70 individuals.

#### 3.3.2 *Girardia tigrina* MFJB

The individuals of *Girardia* and *Dugesia* were collected from under stones a small river near the village Pinggui District, Hezhou City, Guangxi Zhuang Autonomous Region, China (Figure 2B and Table 1). This small river flows through the town. One side of the riverbank was adjacent to several houses. Consequently, there was less vegetation along the riverbank. The rainwater and some domestic wastewater collected in the village were discharged into the small river, making water visibly turbid. Upon microscopic examination, the worms were sorted, and approximately 30 individuals were identified as *Girardia*, all of which were asexual. *G. tigrina* MFJB populations have been maintained under laboratory conditions for three years. The worms reproduced by fission, and the final population size exceeded 100 individuals.

#### 3.3.3 *Girardia tigrina* JXRL

Worms were collected from under stones beneath a small bridge over a stream near the village, Jinxiu Yao Autonomous County, Laibin City, Guangxi Zhuang Autonomous Region, China (Figure 2C and Table 1). The rural primary school is located not far from the stream. On both sides of the stream, there were country paths, with bushes behind them; the section of stream had apparently undergone artificial renovation. Upon microscopic examination, approximately 30 individuals were identified as *Girardia* (asexual). In laboratory conditions, the life cycle of *G. tigrina* JXRL was monitored for six years. The post‐pharyngeal transverse fission occurred continuously, and the strain notably increased in numbers. After about two years in the laboratory, several fissiparous individuals displayed a sexualisation process. However, the sexualized individuals subsequently underwent fission at the post‐pharyngeal region. Thus, they failed to develop the reproductive apparatus and then returned to the fissiparous mode of reproduction. Up to now, the worms increased to approximately 160 individuals.

#### 3.3.4 *Girardia tigrina* XSRK

The individuals of *Girardia* and *Dugesia* were collected beneath submerged stones in a small stream near the entrance of a scenic area in Pingdingshan City, Henan Province, China. The stream was surrounded by dense vegetation and showed no signs of domestic waste or industrial pollution. The water was clear, and domesticated geese were observed feeding and moving within the habitat (Figure 2D and Table 1). Approximately 17 individuals were designated as *Girardia*. Under laboratory conditions, the life cycle of the *G. tigrina* XSRK population was monitored for over three years. During this period, the worms reproduced continuously by fission. To date, the population has increased to approximately 50 individuals.

#### 3.3.5 *Girardia tigrina* QHGY

The individuals were collected from under stones in a river of the Qihe Wetland Park, HeBi City, Henan Province, China. The Wetland Park is a natural reserve, located at the border area between Qibin District and Qi County, and lies in the middle and lower reaches of the Qi River. *Girardia* and *Dugesia* were found at the same collection site (Figure 2E and Table 1). Upon microscopic examination, only six individuals were identified as *Girardia* (asexual). After being kept in the laboratory for about one year, several fissiparous individuals exhibited a sexualisation process. However, these sexualized individuals subsequently underwent fission at the post‐pharyngeal region, failed to develop the reproductive apparatus, and then returned to the fissiparous mode of reproduction. Up to now, the worms increased to approximately 20 individuals.

#### 3.3.6 *Girardia sinensis* SZZ

The worms were collected from beneath stones under a small bridge over a small river close to a village of Huaiji County, Zhaoqing City, Guangdong Province, China. The small river was adjacent to the intersection of two highways. Dense bamboo groves lined both sides of the stream. However, the river water exhibited significant turbidity, and household waste was visible in the water (Figure 2F and Table 1). Approximately 13 individuals were found under stones. Under laboratory conditions, the life cycle of this *G. sinensis* SZZ population was monitored for over two years. The worms continued to reproduce by fission, and to date, the population has increased to approximately 56 individuals.

#### 3.3.7 *Girardia sinensis* HT

Specimens were collected from beneath stones in a river of a village in Bobai County, Yulin City, Guangxi Zhuang Autonomous Region, China. The stream located directly behind a farmhouse received discharged domestic wastewater, leading to evident degradation from prolonged human activity (Figure 2G and Table 1). A total of nine individuals were found under stones at the sampling site. The life cycle of the *G. sinensis* HT population has been monitored under laboratory conditions for five years. During this period, the individuals reproduced exclusively by fission. As of the latest observation, the population size has increased to 45 individuals.

#### 3.3.8 *Girardia sinensis* SSD

Specimens were collected from beneath stones in a river in Pingdingshan, Henan Province, China (Figure 2H and Table 1). The river was adjacent to a nature park and located near a simple temporary prefabricated house. Approximately 50 individuals were identified as asexual *Girardia*. The life cycle of the *G. sinensis* SSD population was monitored under laboratory conditions for two years. Continuous post‐pharyngeal fission was observed, during which the population size increased significantly. As of the latest observation, the population has expanded to approximately 150 individuals.

#### 3.3.9 *Girardia sinensis* HMJ

Specimens of *Girardia* and *Dugesia* were collected from beneath stones in a small river in Lufeng City, Chuxiong Yi Autonomous Prefecture, Yunnan Province, China. The small river flowed through the lushly vegetated mountain valley, and a road passed nearby (Figure 2I and Table 1). Microscopic examination revealed that only ten individuals belonged to *Girardia*, all of which were asexual at the time of collection. The life cycle of this *G. sinensis* HMJ population was monitored for over one year under laboratory conditions. During this period, the individuals did not develop reproductive organs and continued to reproduce asexually. To date, the population has increased to approximately 36 individuals.

In summary, invasive *Girardia* species in China exhibited remarkable environmental adaptability. They were capable of surviving in heavily polluted environments near human settlements and in high water temperatures (above 28°C, see Table 1), while maintaining stable coexistence with *Dugesia* in several minimally disturbed natural zones.

### 3.4 Karyology

**FIGURE 6:**
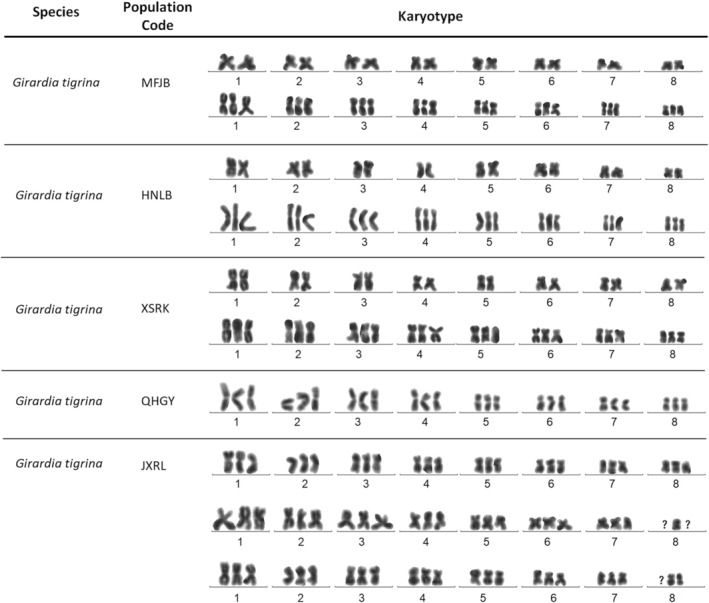
Karyotype analysis of five *G. tigrina* populations (“?” indicates chromosome loss).

All nine *Girardia* populations were analyzed. The karyologyical analysis revealed the karyotypic diversity in *Girardia* from China. The *G. tigrina* MFJB population (8 individuals, 143 metaphase plates were examined) exhibited a mixoploid karyotype with diploid complements of 2*n* = 2*x* = 16 (54 plates) and triploid complements of 2*n* = 3*x* = 24 (86 plates), the remaining 3 plates could not be determined (due to either lack of well dispersed chromosomes or over‐dispersed sets of chromosomes), and all chromosomes were metacentric (Figure 6 and Table 3). All eight specimens exhibited mixoploid chromosome complements. The similar mixoploid karyotype prevailed in *G. tigrina* HNLB and XSRK populations; all specimens exhibited mixoploid chromosome complements with all chromosomes being metacentric. *G. tigrina* HNLB population (8 individuals, 154 metaphase plates were examined) exhibited diploid complements of 2*n* = 2*x* = 16 (39 plates) and triploid complements of 2*n* = 3*x* = 24 (110 plates), remaining 5 plates could not be determined. *G. tigrina* XSRK populaiton (8 individuals, 148 metaphase plates were examined) had diploid complements of 2*n* = 2*x* = 16 (51 plates) and triploid complements of 2*n* = 3*x* = 24 (91 plates), remaining 6 plates could not be determined (Figure 6 and Table 3). In the *G. tigrina* QHGY population, metaphase plates from six randomly selected intact specimens were examined; 151 plates shown triploid karyotype of 2*n* = 3*x* = 24, and the remaining 10 plates could not be determined. All six specimens exhibited triploid chromosome complements, with all chromosomes being metacentric, but the chromosome 6 was at the border between metacentric and submetacentric (Figure 6; Table 3). The *G. tigrina* JXRL population (8 individuals) shown a triploid and aneuploid mosaicism karyotype, with 2*n* = 3*x* = 24 (99 plates), 2*n* = 3*x* = 24–1^8th^ (40 plates), and 2*n* = 3*x* = 24–2^8th^ (24 plates); the remaining 11 plates could not be determined, and all chromosomes being metacentric (Figure 6 and Table 3). Karyotype parameters, including relative length, arm ratio, and centromeric index, are given in Tables S3–S7. Notably, the complex aneuploid (missing 8th chromosome) plus triploid sets with a basic number of 8 metacentric chromosomes represented a karyotype that thus far had not been reported for any other populations of *G. tigrina* .

**FIGURE 7:**
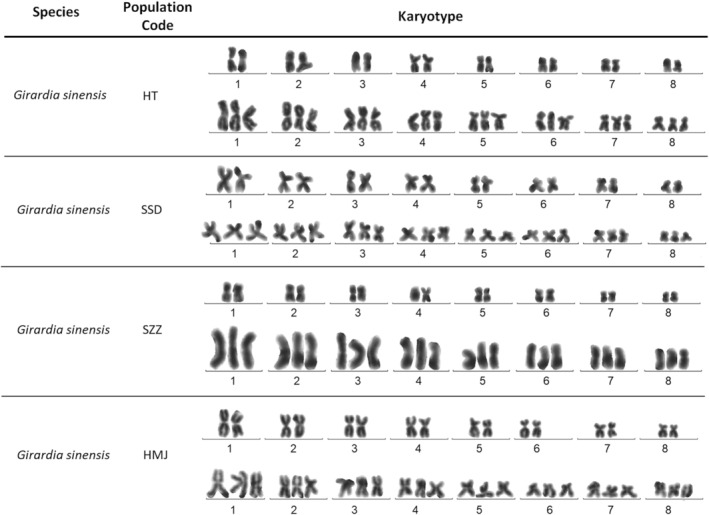
Karyotype analysis of four *G. sinensis* populations.

With respect to the *G. sinensis* , all populations exhibited a mixoploid karyotype with diploid complements of 2*n* = 2*x* = 16 and triploid complements of 2*n* = 3*x* = 24. In *G. sinensis* HT, SZZ and SSD populations, eight, eight and six specimens were examined, respectively; the numbers of diploid metaphase plates were 83, 81 and 50, triploid metaphase plates were 73, 68 and 62, and plates that could not be determined were 11, 6 and 5, respectively. All specimens from these populations exhibited mixoploid chromosome complements with all chromosomes being metacentric (Figure 7 and Table 3). *G. sinensis* HMJ also exhibited mixoploid karyotype, similar to the above populations. Metaphase plates from eight randomly selected intact specimens were examined: 86 plates exhibited diploid karyotype, 94 plates showed triploid karyotype, and remaining 9 plates could not be determined. All eight specimens exhibited mixoploid chromosome complements, with all chromosomes being metacentric, but the chromosome 6 was at the border between metacentric and submetacentric (Figure 7 and Table 3). Karyotype parameters, including relative length, arm ratio, and centromeric index, are given in Tables S8–S11. This study provides the first report of the chromosome karyotype of *G. sinensis* .

## 4 Discussion

### 4.1 Molecular Phylogeny and Genetic Diversity

Our molecular phylogenetic results are generally consistent with those of previous studies (Benítez‐Álvarez, Sluys, et al. 2023 and references therein). The *Girardia* sp. 1 occupies basal positions in our phylogenetic tree. The *G. tigrina* , *G. sinensis* , and *G. dorotocephala* form three distinct clades. To date, relatively few studies have examined interspecific and intraspecific genetic divergence within the genus *Girardia*. However, for *Girardia* sp. 1, the average intraspecific genetic distances were 11.55% for *COI* and 4.13% for *EF1α*, which are substantially higher than that observed in other *Girardia* species examined (Figure 5). This result suggests the possible presence of cryptic diversity within this taxon and indicates that *Girardia* sp. 1 may comprise multiple candidate species. In general, *G. tigrina* exhibits higher intraspecific genetic divergence than *G. sinensis* , indicating a greater level of genetic diversity within *G. tigrina* . This pattern may be related to its broader geographic distribution, viz., Guangdong, Guangxi, Henan, and Yunnan province in China (Figure 1).

### 4.2 Reproductive Modes and Chromosome Karyotype

The first introduction of this genus into Europe occurred when *G. tigrina* was recorded in the UK during the 1920s (Wright 1987). However, only a few cases of sexualized individuals from fissiparous strains of *G. tigrina* have been documented (Stocchino et al. 2019). Most introduced populations of *G. tigrina* reproduce asexually by fission (Sluys et al. 2005). This is consistent with our observation that specimens from China show no external evidence of a gonopore, while signs of asexual reproduction by fission are present. Continued monitoring of this population may help confirm the reproductive strategy of the species in China. In summary, the ongoing degradation of freshwater habitats in China, together with the species' strong capacity for asexual reproduction, indicates the potential for future expansion of *G. tigrina* . Therefore, monitoring its presence and spread in invaded areas and protecting freshwater ecosystems are essential to mitigate potential impacts on native communities.

*Girardia sinensis* , originally described from China, has recently been suggested to be a non‐native species introduced from North America (Chen et al. 2015; Benítez‐Álvarez, Leria, et al. 2023; Benítez‐Álvarez, Sluys, et al. 2023). In the original description, cocoons were reported from Chinese populations, suggesting a potential for sexual reproduction. However, our long‐term monitoring of the studied population revealed no evidence of sexual reproduction. Instead, all individuals reproduced exclusively through fission. Similar observations have been reported from introduced populations in South Africa and Cuba (Trembath et al. 2023; Catalá et al. 2024). This may be due to the fact that the introduced population consists entirely of fissiparous individuals that have lost or suppressed the ability to reproduce sexually (Wittmann et al. 2013).

For *G*. *tirgina*, our karyological analysis reveals that the basic haploid complement of 8 chromosomes is consistent with previous studies (Stocchino et al. 2019). Furthermore, all chromosomes observed in our specimens are metacentric, which also agrees with earlier reports. In contrast, several non‐European populations have been reported to possess karyotypes characterized by seven pairs of metacentric chromosomes and one pair of submetacentric chromosomes (the 6th chromosome set), including a sexual population from Canada (Puccinelli and Deri 1991), sexual and asexual populations from South Brazil, and asexual populations from Japan (Stocchino et al. 2019). Additionally, within the invasive *Girardia* populations in China, we identified triploid (3*n* = 24), mixoploid (diploid and triploid), and aneuploid individuals—the latter exclusively found in the *G. tigrina* JXRL population. To our knowledge, this represents the first report of such karyotypic diversity in *Girardia* from China. The presence of aneuploid and mixoploid individuals in our samples is consistent with observed predominance of asexual reproduction, which may suggest a possible link between karyotypic variation and shifts in reproductive strategies following introduction (Wang et al. 2024). In addition, the chromosomes absence has been only reported in several species of Dugesiidae Ball, 1974, including *D. gonocephala* s. str, (Duges 1830), *D. aethiopica* Stocchino, Corso, Manconi, & Pala, 2002 and *D. musculosa* Chen & Dong, 2024 (Wang et al. 2024 and references therein). However, it is difficult to provide a clear explanation for the above mentioned complex and aneuploid karyotypes (involving the loss of certain chromosomes), although the frequent occurrence of both mixoploidy and aneuploidy in *Dugesia* populations has been postulated to form an adaptive response to environmental pressures.

Moreover, it has been suggested that polyploidization is adaptive in harsh climatic conditions and in extreme environments (Stebbins 1971). Lorch et al. (2016) have reported that chromosome numbers increase with latitude (in three freshwater planarians), with higher latitudes coinciding with harsher climatic conditions. Indeed, high temperatures also represent extreme conditions for freshwater planarians. Accordingly, the presence of polyploid karyotypes in the two invasive *Girardia* species may enhance their adaptability (including tolerance to high temperature) and promote asexual reproduction, thereby facilitating rapid ecological niche occupation in invaded environments.

Taken together, the observed reproductive strategies and karyotype diversity likely contribute to the ecological plasticity and temperature tolerance of *Girardia* species. These traits not only allow populations to persist under suboptimal conditions but also facilitate their rapid expansion in novel habitats.

### 4.3 Ecological Adaptability and Impacts of *Girardia* Invasions

*Girardia* is a genus originally native to South America that spread to North America via natural dispersal and has subsequently become invasive on several other continents (Benítez‐Álvarez, Leria, et al. 2023). Evidence from reproductive characteristics and karyotype variation suggests that *G. tigrina* and *G. sinensis* exhibit considerable ecological plasticity, occupying a wide range of habitats, including artificial aquaria, freshwater streams affected by domestic sewage, heavily human‐impacted environments, and minimally disturbed natural ecosystems (Chen et al. 2015; Stocchino et al. 2019). They also coexist with native *Dugesia* species in some localities, suggesting ecological overlap and potential interspecific competition. Notably, many invaded habitats are characterized by domestic pollution and moderate to high levels of anthropogenic disturbance, conditions that may facilitate the persistence and expansion of fissiparous planarian populations.

In addition to habitat plasticity, physiological tolerance may also contribute to the invasive success of these species. *Girardia tigrina* and *G. sinensis* are generally considered to be most suitable for temperatures between 4°C and 24°C (Vila‐Farré and Rink 2018; Benítez‐Álvarez, Leria, et al. 2023). However, our study recorded populations inhabiting waters ranging from 10°C–32°C and 11°C–30°C, respectively, including specimens collected during summer in southern China. These results suggest a broader thermal tolerance than previously recognized and indicate adaptation to high‐temperature conditions in subtropical regions. Such thermal tolerance may facilitate further range expansion under ongoing climate warming. Furthermore, their predominantly asexual reproductive mode enables rapid population expansion and efficient colonization of available niches. Together with their broad thermal tolerance, these traits raise concerns regarding competitive displacement of native planarian species, alterations in community structure, and broader disruptions to freshwater ecosystem dynamics in China.

Climate change and biological invasions are widely recognized as major drivers of biodiversity and ecosystem change, and climate warming may interact with species' life‐history traits to accelerate biological invasions (Leclerc et al. 2025). Elevated thermal tolerance, coupled with reproductive strategies such as asexual reproduction and karyotype variation, may provide invasive *Girardia* with multiple advantages for colonization across diverse habitats (Svensson et al. 2023). In addition, chromosomal polymorphisms may enhance genomic flexibility, allowing rapid adaptation to novel or stressful environments (Hoffmann et al. 2004). Furthermore, the spread of *Girardia* species exemplifies how freshwater invaders can exploit ecological plasticity and evolutionary adaptability to expand their ranges, particularly under ongoing climate change (Benítez‐Álvarez, Leria, et al. 2023). From a global perspective, these findings highlight the importance of proactive monitoring and management of invasive *Girardia* species, as warming‐driven range expansions may pose increasing threats to native biodiversity and freshwater ecosystem stability in subtropical and temperate regions worldwide (Nimma et al. 2025).

In conclusion, our findings provide a comprehensive baseline of the distribution, reproductive strategies, and environmental adaptations of invasive *Girardia* species, and underscore the urgent need for continued monitoring and effective management to safeguard native biodiversity. Given their broad environmental tolerance and rapid reproductive capacity, together with the ongoing climate warming, proactive management will be essential to limit further spread and mitigate potential ecological disruptions in freshwater ecosystems.

## Author Contributions

**Lei Wang:** data curation (lead), formal analysis (lead), funding acquisition (equal), investigation (equal), writing – original draft (lead), writing – review and editing (equal). **Wei‐Min Ren:** software (supporting). **Xin‐Xin Sun:** data curation (equal), investigation (equal). **De‐Zeng Liu:** writing – review and editing (equal). **Zi‐Mei Dong:** funding acquisition (equal), writing – review and editing (equal). **Guang‐Wen Chen:** funding acquisition (equal), investigation (equal), writing – review and editing (equal).

## Funding

This work was supported by the National Natural Science Foundation of China (Grants 32270501, 32470463, and 32070427), the Foundation for the Key Research Program of Higher Education of Henan Province (25A180017), Postdoctoral Research Project of Henan Province (Grant HN2022136), the Major Public Welfare Project of Henan Province (Grant 201300311700), and by the Puyang Field Scientific Observation and Research Station for Yellow River Wetland Ecosystem, Henan Province.

## Ethics Statement

All handling procedures were strictly compliant with the current Animal Protection Law of China. This study did not involve endangered or protected species. No approvals were required for collections of specimens from the locations in this study. Ethical approvals are not required at Henan Normal University for research conducted on invertebrates such as flatworms used in this study.

## Conflicts of Interest

The authors declare no conflicts of interest.

## Supporting information


**Figure S1:** Molecular phylogenetic tree obtained from ML analysis of the dataset III. Pink circle at nodes indicate support values (pp). *Girardia tigrina* indicated in red, *Girardia sinensis* indicated in blue. Yellow asterisks indicate the current populations of *G. sinensis* in China, while blue asterisks indicate *G. tigrina* in China. Scale bar: substitutions per site.

**Table S1:** Pairwise genetic distances (K2P) based on mitochondrial *COI* sequences among *Girardia* populations and related taxa.

**Table S2:** Pairwise genetic distances (K2P) based on nuclear *EF1α* sequences among *Girardia* populations and related taxa.

**Table S3:** Karyotype parameters (mean values and standard deviations) of *Girardia tigrina* MFJB. m, metacentric.
**Table S4:** Karyotype parameters (mean values and standard deviations) of *Girardia tigrina* HNLB. m, metacentric.
**Table S5:** Karyotype parameters (mean values and standard deviations) of *Girardia tigrina* XSRK. m, metacentric.
**Table S6:** Karyotype parameters (mean values and standard deviations) of *Girardia tigrina* QHGY. m, metacentric.
**Table S7:** Karyotype parameters (mean values and standard deviations) of *Girardia tigrina* JXRL. m, metacentric.
**Table S8:** Karyotype parameters (mean values and standard deviations) of *Girardia sinensis* HT. m, metacentric.
**Table S9:** Karyotype parameters (mean values and standard deviations) of *Girardia sinensis* SSD. m, metacentric.
**Table S10:** Karyotype parameters (mean values and standard deviations) of *Girardia sinensis* SZZ. m, metacentric.
**Table S11:** Karyotype parameters (mean values and standard deviations) of *Girardia sinensis* HMJ. m, metacentric.

## Data Availability

All DNA sequences have been submitted to the National Center for Biotechnology Information (NCBI) GenBank database, and the accession numbers are provided in Table 2, with direct access available through GenBank (https://www.ncbi.nlm.nih.gov/nuccore/). All datasets and statistical analysis results supporting the findings of this study are publicly available as Supporting Information (Tables [Link], [Link], [Link]). Specifically, Tables S1 and S2 contain pairwise genetic distance datasets based on *COI* and *EF1α* sequences, while Tables S3–S11 include karyotype parameters (mean values and standard deviations). Each Supporting Information file includes a ReadMe sheet describing variables, abbreviations, and units to facilitate data interpretation.
