# Supplementary figures and images for: Karyotype Variation and Environmental Adaptation in the Invasive Alien Freshwater Flatworm Girardia in China
Source: Ecol Evol. 2026 Aug 2;16(8):e74095. doi: 10.1002/ece3.74095 (PMC13429354; doi:10.1002/ece3.74095)

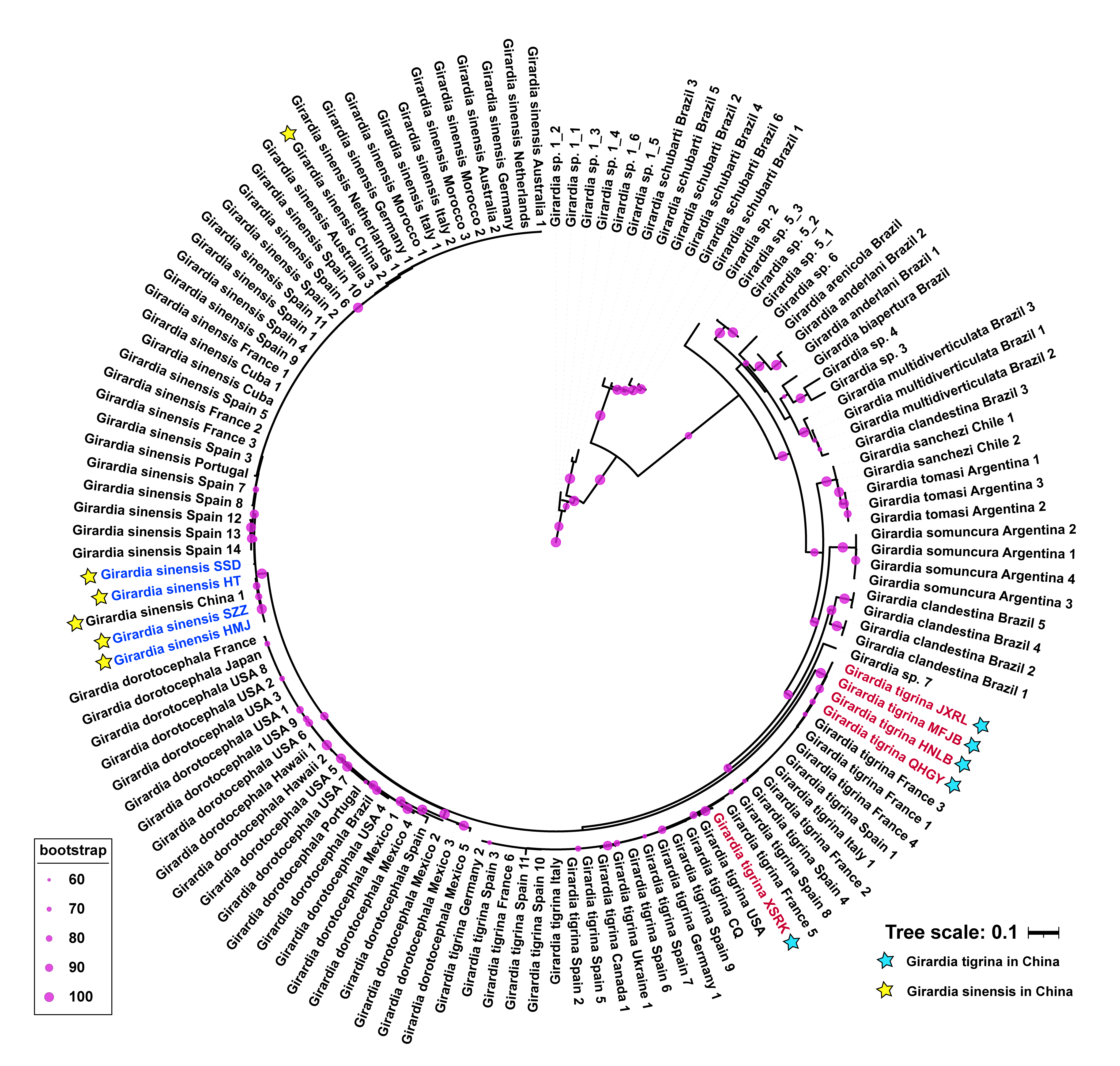

Supplement: Supplementary file 1 — Figure S1: Molecular phylogenetic tree obtained from ML analysis of the dataset III. Pink circle at nodes indicate support values (pp). Girardia tigrina indicated in red, Girardia sinensis indicated in blue. Yellow asterisks indicate the current populations of G. sinensis in China, while blue asterisks indicate G. tigrina in China. Scale bar: substitutions per site. [file ECE3-16-e74095-s001.tif]
