# Supplementary material for: Karyotype Variation and Environmental Adaptation in the Invasive Alien Freshwater Flatworm Girardia in China
Source: Ecol Evol. 2026 Aug 2;16(8):e74095. doi: 10.1002/ece3.74095 (PMC13429354; doi:10.1002/ece3.74095)
Supplement: Supplementary file 4 — Table S3: Karyotype parameters (mean values and standard deviations) of Girardia tigrina MFJB. m, metacentric. Table S4: Karyotype parameters (mean values and standard deviations) of Girardia tigrina HNLB. m, metacentric. Table S5: Karyotype parameters (mean values and standard deviations) of Girardia tigrina XSRK. m, metacentric. Table S6: Karyotype parameters (mean values and standard deviations) of Girardia tigrina QHGY. m, metacentric. Table S7: Karyotype parameters (mean values and standard deviations) of Girardia tigrina JXRL. m, metacentric. Table S8: Karyotype parameters (mean values and standard deviations) of Girardia sinensis HT. m, metacentric. Table S9: Karyotype parameters (mean values and standard deviations) of Girardia sinensis SSD. m, metacentric. Table S10: Karyotype parameters (mean values and standard deviations) of Girardia sinensis SZZ. m, metacentric. Table S11: Karyotype parameters (mean values and standard deviations) of Girardia sinensis HMJ. m, metacentric. [file ECE3-16-e74095-s002.docx]

**Table S3.** Karyotype parameters (mean values and standard deviations) of *Girardia tigrina* MFJB. m: metacentric.

| Chromosome | Relative  length | Arm ratio | Centromeric  index | Chromosome  type |
| --- | --- | --- | --- | --- |
| 1 | 17.16±0.29 | 1.22±0.16 | 45.18±1.70 | m |
| 2 | 15.98±0.36 | 1.22±0.14 | 44.63±1.83 | m |
| 3 | 14.35±0.46 | 1.30±0.15 | 43.94±0.83 | m |
| 4 | 13.01±0.39 | 1.33±0.18 | 43.97±0.86 | m |
| 5 | 11.52±0.33 | 1.35±0.16 | 43.68±1.37 | m |
| 6 | 10.34±0.18 | 1.43±0.12 | 45.05±1.37 | m |
| 7 | 9.46±0.36 | 1.41±0.17 | 43.89±1.44 | m |
| 8 | 8.44±0.14 | 1.42±0.06 | 43.97±1.70 | m |

**Table S4.** Karyotype parameters (mean values and standard deviations) of *Girardia tigrina* HNLB. m: metacentric.

| Chromosome | Relative  length | Arm ratio | Centromeric  index | Chromosome  type |
| --- | --- | --- | --- | --- |
| 1 | 17.76±0.77 | 1.20±0.12 | 45.37±2.03 | m |
| 2 | 15.59±0.43 | 1.26±0.11 | 43.92±1.30 | m |
| 3 | 14.74±0.38 | 1.27±0.13 | 44.41±1.92 | m |
| 4 | 12.62±0.42 | 1.33±0.07 | 43.98±1.21 | m |
| 5 | 11.29±0.33 | 1.31±0.18 | 44.72±1.34 | m |
| 6 | 10.37±0.28 | 1.29±0.21 | 45.81±1.83 | m |
| 7 | 9.49±0.20 | 1.35±0.15 | 43.97±1.34 | m |
| 8 | 8.15±0.54 | 1.43±0.14 | 43.94±1.81 | m |

**Table S5.** Karyotype parameters (mean values and standard deviations) of *Girardia tigrina* XSRK. m: metacentric.

| Chromosome | Relative  length | Arm ratio | Centromeric  index | Chromosome  type |
| --- | --- | --- | --- | --- |
| 1 | 16.88±0.55 | 1.23±0.14 | 44.10±1.44 | m |
| 2 | 15.63±0.40 | 1.24±0.13 | 43.99±1.49 | m |
| 3 | 14.15±0.36 | 1.25±0.14 | 45.15±1.85 | m |
| 4 | 12.73±0.47 | 1.28±0.14 | 44.60±1.60 | m |
| 5 | 11.42±0.32 | 1.23±0.09 | 45.04±1.72 | m |
| 6 | 10.49±0.43 | 1.26±0.14 | 44.54±1.37 | m |
| 7 | 9.54±0.30 | 1.25±0.13 | 44.32±1.78 | m |
| 8 | 8.51±0.26 | 1.28±0.11 | 44.81±1.26 | m |

**Table S6.** Karyotype parameters (mean values and standard deviations) of *Girardia tigrina* QHGY. m: metacentric.

| Chromosome | Relative  length | Arm ratio | Centromeric  index | Chromosome  type |
| --- | --- | --- | --- | --- |
| 1 | 17.19±0.47 | 1.09±0.04 | 47.86±1.19 | m |
| 2 | 15.33±0.16 | 1.12±0.05 | 47.51±1.57 | m |
| 3 | 14.47±0.50 | 1.20±0.10 | 45.19±1.53 | m |
| 4 | 13.13±0.31 | 1.32±0.03 | 43.06±0.62 | m |
| 5 | 11.43±0.54 | 1.23±0.11 | 44.71±1.64 | m |
| 6 | 10.23±0.17 | 1.28±0.19 | 43.12±1.93 | m |
| 7 | 9.41±0.11 | 1.32±0.06 | 43.87±1.13 | m |
| 8 | 8.81±0.30 | 1.27±0.07 | 44.14±1.39 | m |

**Table S7.** Karyotype parameters (mean values and standard deviations) of *Girardia tigrina* JXRL. m: metacentric.

| Chromosome | Relative  length | Arm ratio | Centromeric  index | Chromosome  type |
| --- | --- | --- | --- | --- |
| 1 | 16.25±0.41 | 1.24±0.13 | 44.81±1.49 | m |
| 2 | 14.82±0.08 | 1.22±0.14 | 45.85±1.82 | m |
| 3 | 14.02±0.40 | 1.23±0.06 | 44.93±1.21 | m |
| 4 | 12.92±0.45 | 1.23±0.20 | 46.89±0.87 | m |
| 5 | 11.77±0.35 | 1.30±0.16 | 44.76±1.09 | m |
| 6 | 11.14±0.49 | 1.27±0.06 | 44.27±0.93 | m |
| 7 | 10.53±0.29 | 1.20±0.15 | 45.74±0.97 | m |
| 8 | 9.13±0.54 | 1.28±0.29 | 46.38±0.53 | m |

**Table S8.** Karyotype parameters (mean values and standard deviations) of *Girardia sinensis* HT. m: metacentric.

| Chromosome | Relative  length | Arm ratio | Centromeric  index | Chromosome  type |
| --- | --- | --- | --- | --- |
| 1 | 16.87±0.59 | 1.17±0.07 | 46.53±1.32 | m |
| 2 | 15.12±0.47 | 1.17±0.05 | 46.78±1.15 | m |
| 3 | 13.71±0.28 | 1.19±0.20 | 46.03±1.49 | m |
| 4 | 12.66±0.60 | 1.29±0.31 | 46.32±1.54 | m |
| 5 | 11.89±0.53 | 1.25±0.13 | 45.10±1.45 | m |
| 6 | 10.82±0.61 | 1.26±0.10 | 44.65±1.42 | m |
| 7 | 10.10±0.44 | 1.33±0.17 | 43.42±1.17 | m |
| 8 | 9.00±0.50 | 1.28±0.14 | 45.00±1.47 | m |

**Table S9.** Karyotype parameters (mean values and standard deviations) of *Girardia sinensis* SSD. m: metacentric.

| Chromosome | Relative  length | Arm ratio | Centromeric  index | Chromosome  type |
| --- | --- | --- | --- | --- |
| 1 | 17.53±0.62 | 1.23±0.10 | 45.27±1.46 | m |
| 2 | 14.82±0.45 | 1.30±0.11 | 44.82±1.48 | m |
| 3 | 13.85±0.54 | 1.16±0.13 | 46.76±1.27 | m |
| 4 | 12.45±0.63 | 1.25±0.09 | 44.91±1.30 | m |
| 5 | 11.63±0.50 | 1.21±0.15 | 45.12±1.78 | m |
| 6 | 10.81±0.46 | 1.21±0.05 | 45.64±1.54 | m |
| 7 | 10.05±0.54 | 1.26±0.14 | 43.89±1.06 | m |
| 8 | 8.86±0.41 | 1.16±0.07 | 46.51±1.74 | m |

**Table S10.** Karyotype parameters (mean values and standard deviations) of *Girardia sinensis* SZZ. m: metacentric.

| Chromosome | Relative  length | Arm ratio | Centromeric  index | Chromosome  type |
| --- | --- | --- | --- | --- |
| 1 | 16.93±0.97 | 1.28±0.15 | 43.98±1.19 | m |
| 2 | 15.26±0.94 | 1.16±0.12 | 45.00±1.18 | m |
| 3 | 13.98±0.41 | 1.27±0.16 | 45.23±1.65 | m |
| 4 | 12.76±0.63 | 1.19±0.10 | 44.23±1.39 | m |
| 5 | 11.26±1.01 | 1.24±0.09 | 45.09±1.00 | m |
| 6 | 10.79±0.57 | 1.29±0.16 | 44.57±1.45 | m |
| 7 | 9.69±0.37 | 1.29±0.19 | 45.32±1.36 | m |
| 8 | 9.16±0.51 | 1.21±0.17 | 44.51±1.45 | m |

**Table S11.** Karyotype parameters (mean values and standard deviations) of *Girardia sinensis* HMJ. m: metacentric.

| Chromosome | Relative  length | Arm ratio | Centromeric  index | Chromosome  type |
| --- | --- | --- | --- | --- |
| 1 | 17.64±0.50 | 1.24±0.10 | 46.01±2.46 | m |
| 2 | 14.79±0.33 | 1.26±0.13 | 46.59±0.79 | m |
| 3 | 13.56±0.49 | 1.21±0.13 | 44.85±1.83 | m |
| 4 | 12.79±0.33 | 1.25±0.16 | 44.94±2.54 | m |
| 5 | 11.90±0.54 | 1.27±0.09 | 45.26±1.39 | m |
| 6 | 10.78±0.27 | 1.37±0.07 | 42.60±1.81 | m |
| 7 | 9.66±0.40 | 1.30±0.16 | 45.16±2.38 | m |
| 8 | 8.88±0.68 | 1.26±0.10 | 43.58±1.04 | m |
